# *Prunus mume* Concentrate and Ca^2+^ Dual Cross-Linking Facilitate Sodium Alginate/Carboxymethyl Chitosan/Gelatin Microcapsules for Probiotic Encapsulation

**DOI:** 10.3390/ijms27094141

**Published:** 2026-05-06

**Authors:** Tenglong Miao, Ni An, Huhu Wang, Chuang Zhang, Xin Rui, Qiuqin Zhang, Xinglian Xu

**Affiliations:** 1 Sanya Institute of Nanjing Agricultural University, Sanya 572024, China; 2 College of Food Science and Technology, Nanjing Agricultural University, Nanjing 210095, China

**Keywords:** probiotics, plant concentrate, microcapsule, digestive characteristics, dual cross-linking

## Abstract

This study presents a novel dual cross-linking method using *Prunus mume concentrate* (PMC) as a source of H^+^ and Ca^2+^ to enhance polysaccharide (sodium alginate/carboxymethyl chitosan/gelatin) microcapsule formation. The structure and release characteristics of microcapsules were influenced more by PMC pH than by its concentration. SEM results showed that as the pH decreased, the microcapsules had a more compact structure. The FTIR results showed that acid enhances hydrogen bonding and electrostatic interactions within the polysaccharide, leading to more stable microcapsule structures. XRD patterns showed that acid enhanced the stability of the polysaccharide crystal structure. Microcapsules significantly increased viable counts by 1 log(CFU/mL) in simulated gastric fluid (SGF) and 1.25 log(CFU/mL) in simulated intestinal fluid (SIF) after 3 h of digestion. This study provides a basis for investigating the dual cross-linking of natural plant concentrates and Ca^2+^ construction of polysaccharide microcapsules to enhance probiotic resistance.

## 1. Introduction

Lactic acid bacteria (LAB) are widely recognized for their health benefits, but their survival during gastrointestinal transit remains a major challenge, especially in gastric fluid, making it difficult for them to colonize the intestine through gastric fluid, which limits their probiotic function in the human body [1]. Therefore, improving the survival rate of probiotics during gastrointestinal digestion is critical. Nowadays, probiotics encapsulation is an important method to effectively maintain their vitality and stability. Encapsulation is a technique that uses natural biomolecule materials such as polysaccharides, proteins, and lipids as barriers to protect probiotics, in order to resist the erosion of gastric acid and be degraded by intestinal microorganisms, achieving the release and colonization of the encapsulated probiotics in the intestine [2].

Sodium alginate (SA), a common polysaccharide, easily forms egg box dimer with metal ions through its α-L-guluronic acid to form gel microcapsules, of which Ca^2+^ is the most stable [3]. However, SA is easily digested in the acidic environment of gastric fluid, which will lead to the early release of probiotics from the microcapsules. Microcapsules prepared from SA often have a porous structure, with a width of generally 5–200 nm, which cannot prevent gastric acid from diffusing into microcapsules, limiting their protective effect on probiotics [4,5,6,7]. Carboxymethyl chitosan (CMCS) is a typical zwitterionic polysaccharide polymer modified by substituting some -NH_2_ and -OH in chitosan with -CH_2_COOH [8]. CMCS has hemostatic, wound healing-promoting, and antibacterial effects, and also has ideal water solubility. Due to the -NH_2_ in CMCS, it dissolves in weakly alkaline environments but does not dissolve in acidic environments, which can compensate for the deficiency of SA digestion under gastric acid conditions. The -NH_2_ pKa value is about 6.5 in CMCS, while the -COOH pKa value is about 3.5 in SA, indicating that both functional groups are protonated when the pH value is less than 3.5 [9]. Therefore, by providing an acidic environment, the physical cross-linking of CMCS and SA can be promoted. Ca^2+^ facilitates the chemical cross-linking of SA, forming a dual cross-linking effect, which may lead to a more stable structure.

Compared with traditional modification using chemical reagents, organic acids used to construct microcapsules have better safety. Scholars have used the acidic conditions provided by citric acid to protonate the -COOH in SA and -OH, -NH_2_ in CMCS to form hydrogen bonds, resulting in microcapsules with good stability under low acid conditions [10]. Due to the easy formation of more interaction forces and better stability of polysaccharide protein complexes, adding proteins to a single polysaccharide system can improve the stability of microcapsules, such as the formation of stable hydrogen bonds between -NH_2_ in gelatin (GE) and -COOH in SA, thereby enhancing the viscosity and stability of the network structure [11,12]. Many plants contain many organic acids, such as *Crataegus* pinnatifida Bunge, *Schisandra* chinensis (Turcz.) Baill., and *Prunus mume* Siebold & Zucc. [13,14,15]. However, many plant extracts are rich in organic acids and contain some other beneficial components, which have not been applied in the preparation of microcapsules. Therefore, organic acids from plant sources are used to dual cross-linking with Ca^2+^ to prepare microcapsules for probiotics encapsulation, improving their survival ratio in the human digestive tract and better exerting their probiotic effects.

*Prunus mume* Sieb. et Zucc. is a unique fruit tree species native to China, known for its high content of citric acid in its fruits. The titratable acidity (calculated by citric acid equivalent) of *Prunus mume* concentrate (PMC), which is made by concentration and processing, is usually between 8% and 12%, and the maximum is about 15%. It is an excellent natural pH regulator in the food industry, and it contains a unique functional substance with physiological activity—mumefural [16]. This study aims to promote green production by designing a dual cross-linking with Ca^2+^ and PMC construction of polysaccharide microcapsules that can effectively improve the protective effect of microcapsules on probiotics. Sodium alginate carboxymethyl chitosan gelatin (SA-CMCS-GE) complex was used to encapsulate *Lactiplantibacillus plantarum* JB-1. Microcapsules were prepared using cross-linking solutions of different pH and concentrations of *Prunus mume* concentrate, and the encapsulation efficiency, structure, swelling characteristics, stability, and protective effects of the microcapsules were measured.

## 2. Results and Discussion

**Figure 1:**
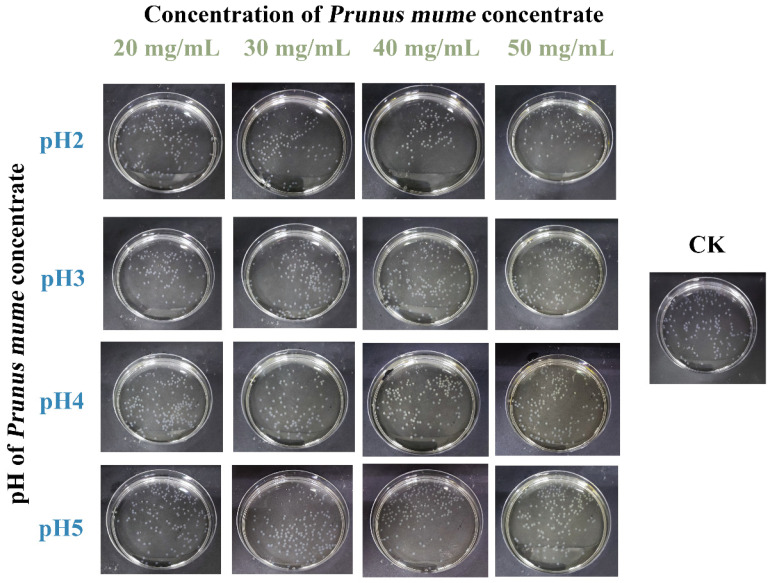
Images of different microcapsules. In the CK group, the microcapsules without the addition of *prunus mume* concentrate were used as the blank control.

The protective function of microcapsules is influenced by their size; smaller diameters generally offer better protection [17]. The various microcapsules prepared in this experiment are shown in Figure 1, which exhibited regular spherical shapes and smooth opaque white surfaces. The various microcapsule diameters were between 1.8 and 2.33 mm (Table 1). As the concentration of PMC in the cross-linking solution increased, there was no significant difference in microcapsule diameter at the same pH (*p* > 0.05). However, in the cross-linking solution with the same concentration of PMC, as the pH gradually decreased, the diameter of the microcapsules significantly decreased (*p* < 0.05). Compared with the CK group, there was no significant difference (*p* > 0.05) in the formation of microcapsules in the pH 4 and pH 5 cross-linking solutions, but there was a significant difference (*p* < 0.05) in the formation of microcapsules in the pH2 and pH3 cross-linking solutions. Microcapsule formation is primarily attributed to the “egg box” model formed by the electrostatic interaction between Ca^2+^ and -COOH from the SA-CMCS-GE mixture [18]. At the same time, the H^+^ provided by the low pH environment would form hydrogen bonds with -CH_3_ and other functional groups in the SA-CMCS-GE mixture [10]. Compared with the ionic cross-linking of Ca^2+^ and the ionic cross-linking formed by hydrogen bonds used in traditional microcapsule formation, this study combined two cross-linking methods to achieve dual cross-linking, further intensifying the formation of hydrogen bonds and electrostatic interactions in microcapsules. The microcapsules prepared by a low pH cross-linking solution had a smaller diameter.

**Figure 2:**
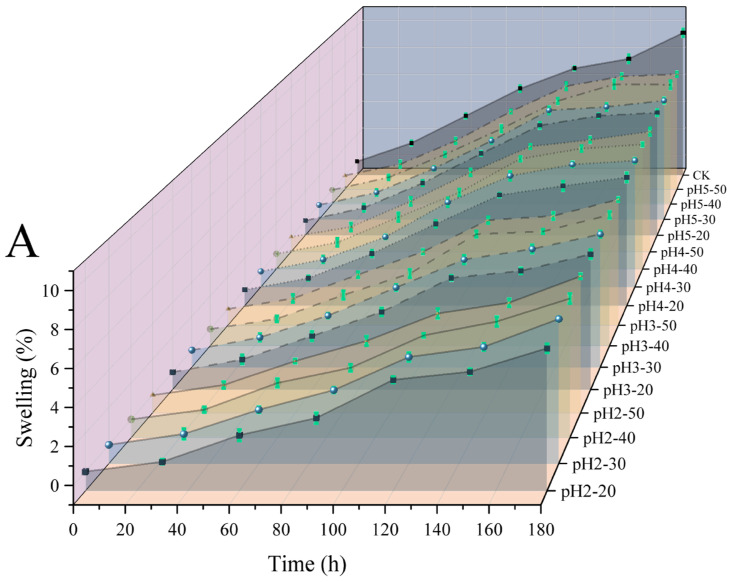

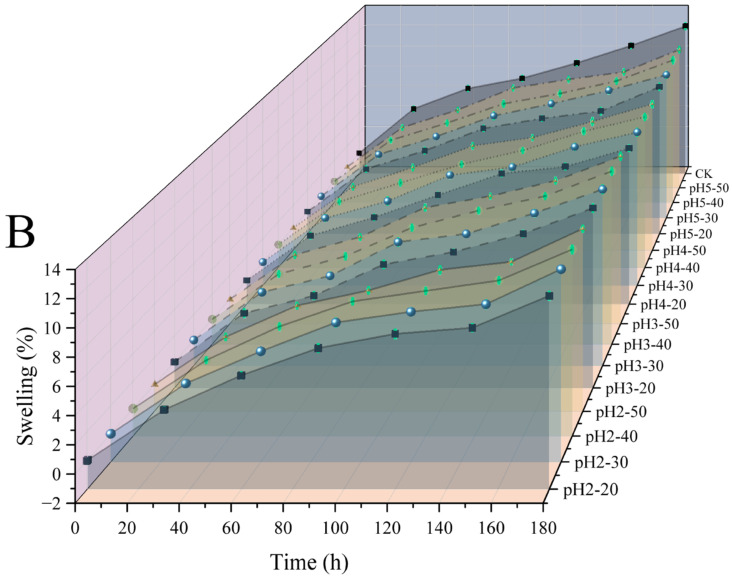
Swelling ratio of microcapsules in SGF (**A**) and SIF (**B**).

The release characteristics of microcapsules largely depend on their swelling properties. Microcapsules had low swelling in SGF and high swelling in SIF, which enabled them to protect probiotics in SGF and release probiotics in SIF. Figure 2 shows the swelling ratio of microcapsules in simulated gastric and intestinal fluids. After a period of swelling, it is evident that in SGF (pH2.5), the swelling ratio of microcapsules was significantly lower than in SIF (pH7). In SGF (pH2.5), the swelling ratio of microcapsules first increased rapidly and finally reached saturation. Within the first 120 min, all microcapsules swelled rapidly, and at the same time, the swelling ratio of all microcapsules was lower than that of the CK group. This indicated that the microcapsules constructed in this study had better stability under acidic conditions, prevented premature release of probiotics, and better protected probiotics. The swelling ratio of microcapsules was closely related to the interactions between their functional groups [19]. In SGF, the swelling ratio of microcapsules was less than 10, which was limited by the hydrogen bonding between -COOH and -OH and the electrostatic interaction between -NH^3+^ and -COOH in the microcapsules. In contrast, in SIF, the swelling ratio of microcapsules continuously increased rapidly. However, at the same time, there was no significant difference in the swelling ratio of all microcapsules compared to the CK group. This may be because the acidic environment of the cross-linking solution changed the interaction force of the internal groups of microcapsules, but did not change the interaction force of the groups under weakly alkaline conditions. The -COOH in microcapsules gradually ionized into -COO, and due to the electrostatic repulsion between -COO, the microcapsules significantly swelled, leading to network expansion, which contributed to the microcapsules releasing probiotics [20]. The microcapsules prepared by dual cross-linking in this experiment have smaller swelling in SGF, better protection of probiotics, a high swelling ratio in intestinal fluid, and ensured good release properties.

**Figure 3:**
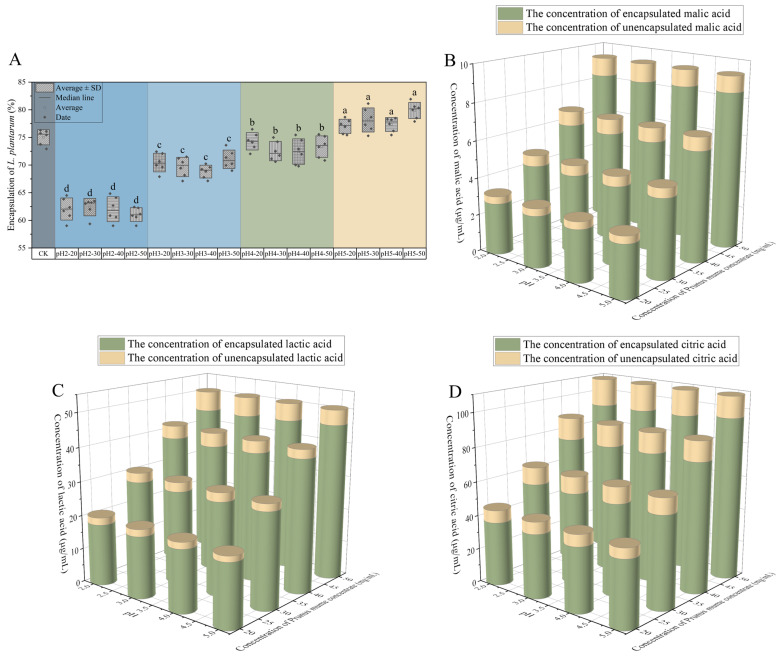
Encapsulation efficiency (EE) of *L. plantarum* (**A**) in microcapsules. The concentration of encapsulated malic acid (**B**), lactic acid (**C**) and citric acid (**D**). Note: Lowercase letters indicate significant differences (*p* < 0.05) between different pH values at the same concentration of PMC.

The encapsulation effect of microcapsules is reflected by the encapsulation efficiency (EE) of probiotics. The higher the EE of microcapsules, the more probiotics could be escorted to the intestine [21,22]. As shown in Figure 3, at the same pH, different concentrations of PMC of microcapsules had no significant difference (*p* > 0.05) in the EE of *L. plantarum*. However, at the same concentration of PMC, there was a significant difference (*p* < 0.05) in the EE of *L. plantarum* between different pH levels. As the pH decreased, the EE of *L. plantarum* decreased significantly. According to Table 2, as the pH decreased, the diameter of microcapsules decreased significantly. A smaller diameter would typically suggest higher EE, but low pH may reduce bacterial viability, possibly because low pH would cause the death of some *L. plantarum*. There was no significant difference (*p* > 0.05) in the EE of *L. plantarum* between the pH 4 group and the CK group (75.03 ± 1.37%), and the EE of *L. plantarum* at pH 5 was significantly (*p* < 0.05) higher than the CK group, while the EE of *L. plantarum* at pH 2 and 3 was significantly (*p* < 0.05) lower than the CK group. Based on the above results, the concentration of PMC has no effect on the EE of *L. plantarum*. Although low pH helped the microcapsules form a smaller appearance and a more stable structure internally, the large amount of H^+^ brought by low pH may have adverse effects on *L. plantarum*, reducing its EE. The pH effects on bacterial viability are speculative. The highest EE of *L. plantarum* was 79.87 ± 1.46% when the cross-linking solution was pH5 and 50 mg/mL PMC.

Figure 3 lists the EE of organic acids in microcapsules. At the same concentration of PMC, as the pH increased, the EE of lactic acid, malic acid, and citric acid gradually decreased. This might be because lactic acid, malic acid, and citric acid act as H^+^ donors, and under low pH conditions, they are more likely to dissociate in the cross-linking solution, thereby adhering to the interior of microcapsules and improving their EE. At the same pH, as the concentration of PMC increases, the EE of malic acid and citric acid showed a gradually decreasing trend, while lactic acid showed a gradually increasing trend. This may be because malic acid and citric acid are respectively binary and ternary acids, while lactic acid is a monobasic acid. The stronger acidity of malic acid and citric acid allows them to accumulate in large quantities in microcapsules at low concentrations of PMC. However, as the concentration of PMC increased, it became more difficult to accumulate a large amount of malic acid and citric acid inside the microcapsules, and lactic acid had lower acidity as a monobasic acid. Therefore, when the concentration of PMC increased, more lactic acid could accumulate inside the microcapsules. The highest EE of malic acid was at pH2 cross-linking solution, with 20 mg/mL PMC of 13.38%. The highest EE of citric acid was at pH2 cross-linking solution, with 30 mg/mL PMC of 16.33%. The highest EE of lactic acid was at pH2 cross-linking solution, with 50 mg/mL PMC of 11.87%.

**Figure 4:**
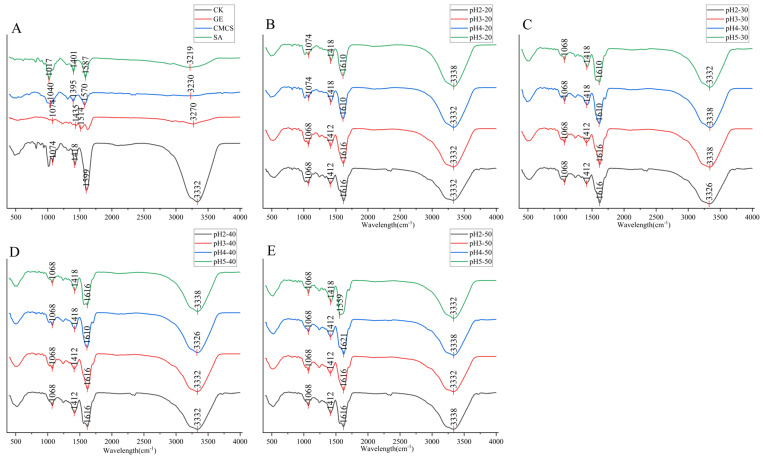
FT-IR spectra recorded from GE, CMCS, SA and CK (**A**), 20 mg/mL *Prunus mume* concentrate (**B**), 30 mg/mL *Prunus mume* concentrate (**C**), 40 mg/mL *Prunus mume* concentrate (**D**), 20 mg/mL *Prunus mume* concentrate (**E**).

The FT-IR spectrum of microcapsules is shown in Figure 4 to analyze the interaction forces between internal functional groups of microcapsules. The peak values of the C=O characteristic peak at 1587 cm^−1^, the O-H characteristic peak at 1401 cm^−1^, and the C-O characteristic peak at 1017 cm^−1^ in SA migrate to higher wavenumbers of 1605 cm^−1^, 1420 cm^−1^, 1074 cm^−1^ in microcapsules, respectively; The C=O characteristic peak at 1570 cm^−1^, the CH_3_ characteristic peak at 1395 cm^−1^, and the C-O characteristic peak at 1040 cm^−1^ in CMCS also shifted to 1605 cm^−1^, 1420 cm^−1^, 1074 cm^−1^ in microcapsules, indicating hydrogen bonding interactions between microcapsules [10]. The C=O characteristic peak of GE’s amide I band shifted from 1627 cm^−1^ to 1605 cm^−1^, indicating the existence of electrostatic interactions between polymers. At the same time, the characteristic peak of GE’s OH at 3270 cm^−1^ and SA/CMCS’s OH at 3268 cm^−1^ both shifted towards 3333 cm^−1^, indicating the formation of hydrogen bonds inside the microcapsules. The above results confirmed that hydrogen bonding and electrostatic interactions were the main forces involved in the formation of microcapsules [23]. The peak values of the characteristic peaks of various microcapsules were generally located at 1068 cm^−1^, 1412 cm^−1^, 1616 cm^−1^, 3338 cm^−1^, 1420 cm^−1^, 1074 cm^−1^, which were higher than the peak values of the characteristic peaks of the CK group at 1074 cm^−1^, 1420 cm^−1^, 1605 cm^−1^, 3332 cm^−1^. This indicated that the H^+^ provided under acidic conditions promotes hydrogen bonding and electrostatic interactions between polymers, making the structure of microcapsules more stable [9,10,24]. However, there was no significant difference in the peak values of the characteristic peaks of various microcapsules, which may be due to the fact that the molecular forces inside the microcapsules have reached their maximum under pH5 acidic conditions. The pH decrease does not promote the formation of more stable structures in the microcapsules. In summary, the FTIR results not only confirmed that hydrogen bonding and electrostatic interactions were the main forces involved in the formation of SA/CMCS/GE microcapsules, but also demonstrated that acid helped to enhance the intermolecular forces of microcapsules, resulting in a more stable structure.

**Figure 5:**
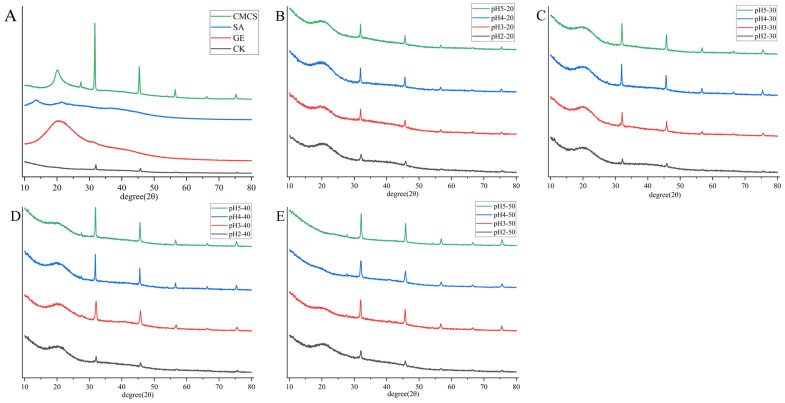
XRD spectra recorded from GE, CMCS, SA and CK (**A**), 20 mg/mL *Prunus mume* concentrate (**B**), 30 mg/mL *Prunus mume* concentrate (**C**), 40 mg/mL *Prunus mume* concentrate (**D**), 20 mg/mL *Prunus mume* concentrate (**E**).

The XRD of microcapsules is shown in Figure 5 to characterize the crystalline properties of polymers or compounds [25,26]. A characteristic broad peak was observed at 20° for SA, indicating that SA is in a semi-crystalline state. Characteristic peaks were observed at 31.6°, 45.5°, 55.2°, 65.12°, and 75.3° for CMCS, indicating that CMCS has a crystalline structure; GE observed a characteristic broad peak at 12.7°, indicating that GE is in a semi-crystalline state [27,28]. The XRD patterns of all microcapsules retained the characteristic peaks of CMCS, as well as the broad peaks of GE and SA, indicating that all microcapsules were in a semi-crystalline state. The sharper the peak height of the XRD pattern, the better the crystallinity of the compound. Compounds with better crystallinity have ordered lattice structures, making them more stable at high temperatures [29]. As the pH of the cross-linking solution increased, the peak height of the microcapsules became sharper, indicating better crystallinity of the microcapsules. This may be due to the electrostatic interactions between -NH_2_ and -COOH between SA, CMCS, and GE, as well as the strengthening of hydrogen bonds under acidic conditions, resulting in a decrease in peak height [30]. The microcapsules prepared using pH4 and pH5 cross-linking solutions had higher peak heights than the CK group, indicating better stability. In summary, changing the pH of the cross-linking solution could alter the XRD properties of microcapsules, with the microcapsules prepared at pH 4 showing the best stability. The XRD results primarily highlight the crystal structure of the polymer, whereas our polymer comprises a significant amount of semi-crystalline or amorphous structures. Although its crystal structure is more stable, the overall stability of the microcapsule still needs to be comprehensively evaluated based on other indicators.

**Figure 6:**
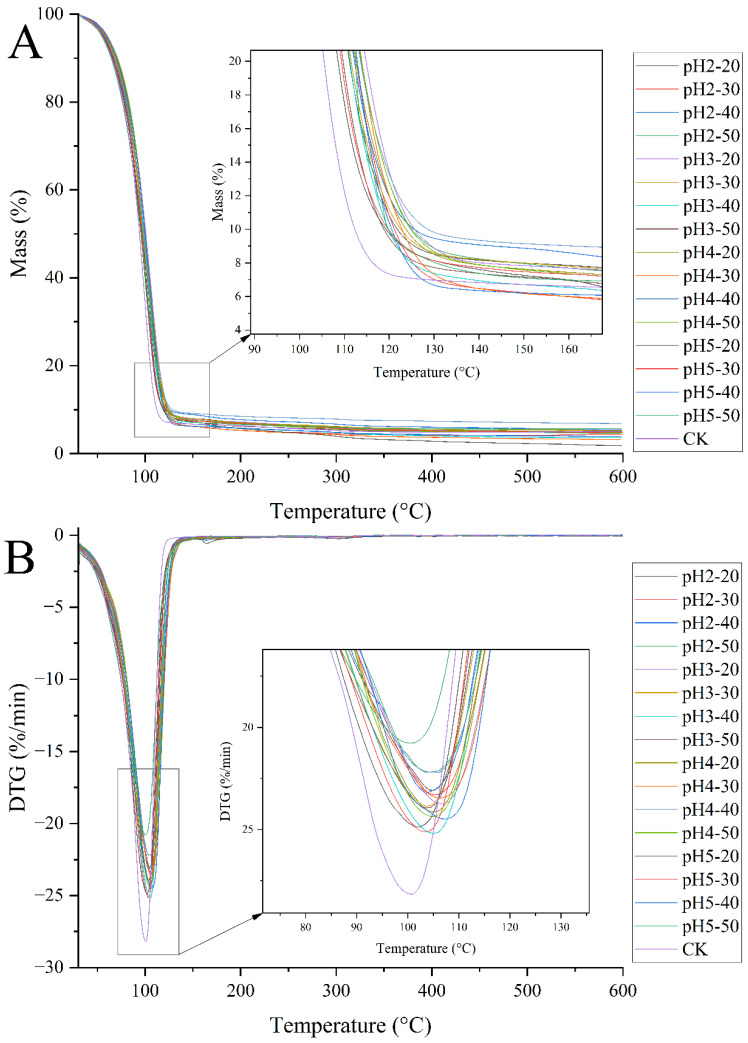
Thermogravimetric (TG) curve (**A**) and differential thermogravimetric (DTG) curve (**B**) of microcapsules.

TGA is an important tool for evaluating the thermal stability of compounds [31,32]. The thermogravimetric (TG) curves of microcapsules are shown in Figure 6A, which characterizes the change in mass of the sample with increasing temperature during the set temperature rise process. From Figure 6A, A single major degradation stage was observed between 120–125 °C. The thermogravimetric differential (DTG) curves of microcapsules are shown in Figure 6B (dm/dt curve, a curve obtained by taking a derivative of each point on the TG curve with respect to the time coordinate), used to represent the change in the rate of mass change with increasing temperature. Its peak point represents the temperature at which the mass change rate is the fastest [33]. From Figure 6B, it can be seen that there is no significant difference in the degradation temperature of all microcapsules between 100–110 °C. Therefore, it can be concluded that the thermal stability of microcapsules is independent of pH and the concentration of PMC, and is only related to the material. Because the basic materials used to prepare microcapsules are the same, there is no significant difference in thermal stability between the microcapsules. Since there is no significant correlation between the concentration of PMC and the characteristics of microcapsules, the pH of PMC is related to the microcapsules’ characteristics. Next, microcapsules were prepared using a fixed concentration of 50 mg/mL of PMC and cross-linking solutions with different pH levels for further study.

**Figure 7:**
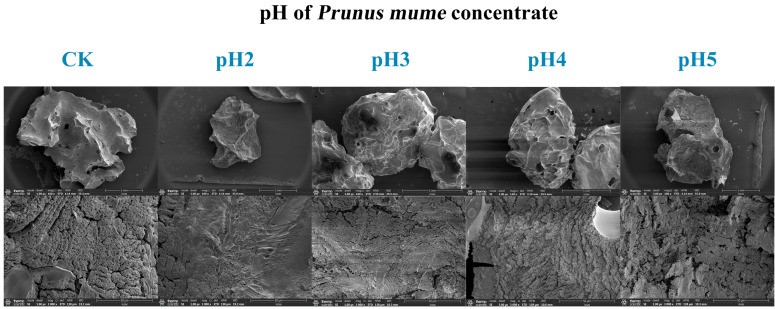
SEM of 50 mg/mL PMC with different pH levels of microcapsules (The magnification of the photos in the first row is 100×, and that of the photos in the second row is 3000×.).

From the SEM of microcapsules, it could be seen that the spherical structure of the microcapsule particles was intact, with many pores on the surface and an uneven surface (Figure 7). As the pH of the cross-linking solution decreased, the pores of the microcapsules also decreased and became smaller. Among them, the microcapsules prepared with pH2 cross-linking solution had the smallest and fewest pores, while the microcapsules prepared with pH5 cross-linking solution had no significant difference in pores compared to the CK group. This indicated that the dual cross-linking method significantly changed the microstructure of the microcapsules, making them have a better structure to protect probiotics, which was consistent with the macroscopic observation results of microcapsules (Table 2). A low pH cross-linking solution made microcapsules have a tighter and smaller structure. As the pH of the cross-linking solution decreased, the surface of the microcapsules also became smoother and tighter (Figure 7), indicating that the microcapsules prepared by the dual cross-linking method had a relatively stable structure and stronger ability to resist adverse environments, which can better protect probiotics and reduce probiotic damage [34]. In summary, changing the pH of the cross-linking solution to prepare microcapsules not only altered the macroscopic and microscopic structures of the microcapsules but also resulted in smaller and more compact structures, which may enhance the protective effect of microcapsules on probiotics.

**Figure 8:**
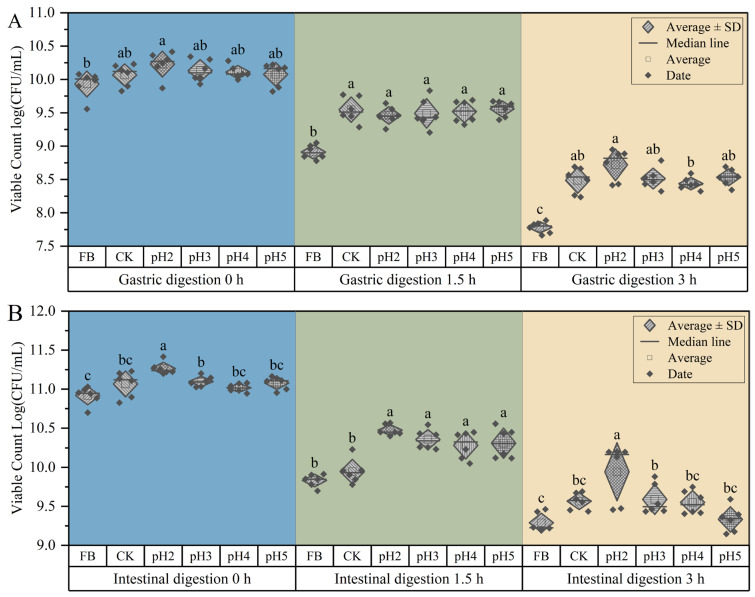
Survival ratio of microcapsules in SGF (**A**) and SIF (**B**). Note: Lowercase letters indicate significant differences between groups at the same digestion time (*p* < 0.05).

Whether microcapsules can help probiotics overcome the low acid environment of gastric digestion is an important indicator for evaluating the protective effect of microcapsules [35]. Due to the fact that the concentration of PMC had little effect on the properties of microcapsules, this experiment studied the viable count in free bacteria, and microcapsules prepared at different pH levels during 3 h SGF digestion. The results are shown in Figure 8A. The results showed that after 1.5 h SGF digestion, the viable count in free bacteria significantly decreased by about 1 log (CFU/mL) (*p* < 0.05), while the viable count in microcapsules decreased by about 0.75 log(CFU/mL), but there was no significant difference between each microcapsule (*p* > 0.05). After 3 h SGF digestion, the viable count in free bacteria decreased significantly by about 1.25 log (CFU/mL) (*p* < 0.05), while the viable count in microcapsules was significantly higher than that of free bacteria (*p* < 0.05), indicating that microcapsules had a good protective effect on *L. plantarum* during the gastric digestion stage. Microcapsules prepared with pH2 cross-linking solution had the most viable count at 8.72 log(CFU/mL), significantly higher than other microcapsules (*p* < 0.05). This may be due to the tighter structure of microcapsules prepared with pH2 cross-linking solution, which provided better protection for *L. plantarum*, or it may be due to the lower acidity environment during microcapsule preparation, enhancing *L. plantarum* resistance to SGF [23,36].

Microcapsules also need to protect probiotics in the intestine to cope with adverse environments such as intestinal trypsin and high bile salts, allowing probiotics to remain in the intestine for sufficient time to colonize [37,38]. This experiment investigated the viable count in free bacteria and microcapsules prepared at different pH levels during 3 h SIF digestion. The results are shown in Figure 8B. Compared to digestion in SGF, *L. plantarum* in SIF had a higher viable count. The results showed that after 1.5 h intestinal digestion, there was no significant difference in the viable count in free bacteria and the viable count in the CK group, both of which decreased by about 1 log(CFU/mL). However, the viable count in microcapsules prepared with different pH decreased by about 0.5 log(CFU/mL), which was significantly lower than the decrease in the viable count in the previous two groups (*p* < 0.05). This indicated that microcapsules prepared with different pH cross-linking solutions can improve the protective effect of *L. plantarum* in intestinal digestion, but there was no significant difference between each microcapsule (*p* > 0.05). After 3 h intestinal digestion, the viable count decreased significantly (*p* < 0.05) by about 1 log(CFU/mL), while the viable count encapsulated in microcapsules was significantly higher than free bacteria (*p* < 0.05). At this time, the microcapsules prepared with pH2 cross-linking solution encapsulated the highest viable count, which was 9.94 log(CFU/mL), significantly higher than other microcapsules (*p* < 0.05). In summary, the microcapsules prepared by the dual cross-linking method significantly increased the viable count in SIF, and the protective effect was better than that of the CK group. The microcapsules prepared with the cross-linking solution at pH2 had the best protective effect.

**Figure 9:**
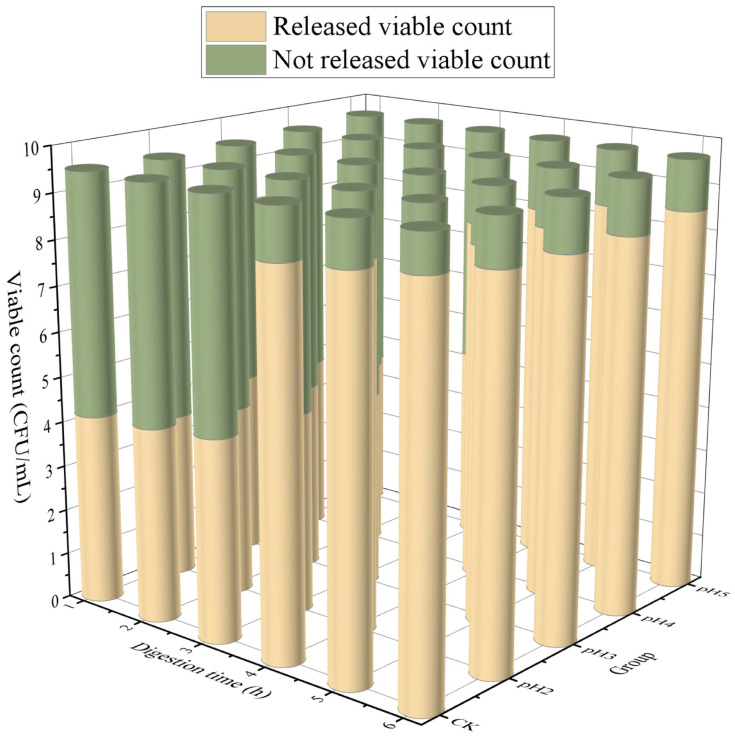
Released viable count in simulated digestive fluid of microcapsules.

This experiment established a simulated human digestive system for 3 h SGF digestion and 3 h SIF digestion to evaluate the release characteristics of microcapsules in the human simulated digestive system. The viable count and release ratio of probiotics from microcapsules are shown in Figure 9. Due to the insufficient amount of data, we did not conduct modeling but instead performed a simple statistical analysis. The experimental results showed that the microcapsule released only a little viable count of about 3.60–4.46 log(CFU/mL). After 5 h of digestion, the disintegration of microcapsules was achieved, and the released viable count reached 8.33–8.69 log(CFU/mL). This was mainly related to the swelling characteristics of the microcapsules in the digestive fluid. The R-COOH in the microcapsules was protonated in the SGF (pH2.5), resulting in less electrostatic repulsion and enhanced hydrogen bonding force, making the network structure in the microcapsules more stable and less likely to be broken. When the intestinal digestion stage, the microcapsules rapidly swelled, and a large number of probiotics encapsulated in the microcapsules were gradually released. This was mainly because the sudden pH increase in the intestinal fluid caused the R-COOH in the microcapsules to dissociate into negatively charged R-COO^−^. The electrostatic repulsion between R-COO^−^ would lead to the expansion of the gel network [39,40].

During the gastric digestion stage, the released viable count was in descending order of pH2, pH4, CK, pH3, and pH5. It could be concluded that the improved microcapsules have a lower released viable count than the CK group in SGF. Among them, the microcapsules prepared with pH2 cross-linking solution had a higher released viable count, which may be due to their better resistance in SGF. The microcapsules prepared with other cross-linking solutions may have a higher released viable count, but more of them died. During the intestinal digestion stage, the released viable count was in the order of pH2, pH5, CK, pH3, and pH4. From this, it could be concluded that the microcapsules prepared by dual cross-linking have a lower released viable count in SIF compared to the CK group. Among them, the microcapsules prepared with pH2 cross-linking solution release more viable bacteria, possibly because their modification degree under acidic conditions was much greater than that of other microcapsules. At the same time, the released viable count of all microcapsules in SIF reached 7 log(CFU/mL). Microcapsules prepared by dual cross-linking could deliver enough live probiotics to the intestine, allowing probiotics to exert probiotic effects. In summary, microcapsules prepared with pH2 cross-linking solution could better protect probiotics in SGF and release probiotics in SIF.

## 3. Materials and Methods

### 3.1. Culture Conditions of Bacterial Strains

*Lactiplantibacillus plantarum* JB1 was obtained from the culture collection of Nanjing Agricultural University, Nanjing, China [41]. *L. plantarum* JB1 was cultured in de Man, Rogosa and Sharpe (MRS) medium (Qingdao Hope Bio Technology Co., Ltd., Qingdao, China).

### 3.2. Materials

*Prunus mume* concentrate (PMC) was purchased from Nanjing Longlijia Agricultural Development. Sodium alginate (SA, β-D-mannuronic:α-L-guluronic (M/G) = 1:2, molecular weight: 140–150 KDa, CAS: 9005-38-3), carboxymethyl chitosan (CMCS, molecular weight: 240 KDa degree of deacetylation > 80% CAS: 83512-85-0), commercial type A gelatin (GE, CAS: 9000-70-8, 20–250 KDa) from porcine skin and Calcium chloride anhydrous (CAS: 1718-84-6) were bought from Shanghai Macklin Biochemical Co., Ltd. NaOH and HCl were bought from Guangdong Guanghua Sci-Tech Co., Ltd. Acetonitrile, methanol and formic acid (HPLC) were procured from TEDIA (TEDIA COMPANY, INC., Fairfield, OH, USA). All simulated digestive fluid reagents were purchased from Sinopharm Chemical Reagent Co., Ltd. (Shanghai, China). Gastric protease (P7125, >400 U/mg), gastric mucin (M2378), pancreatin (B8631), and bile salt (P7545) were from Sigma-Aldrich, Inc (Burlington, MA, USA).

### 3.3. Preparation of Gel Complex, Cross-Linking Solution and Simulated Digestive Fluid

Preparation of SA-CMCS-GE complex: SA solution (2.5%, *w*/*v*), CMCS solution (2.5%, *w*/*v*), and GE solution (2.5%, *w*/*v*) were prepared using sterile distilled water and stirred for 2 h to mix evenly. SA, CMCS, and GE solutions were mixed in a ratio of 1:1:1 (*v*/*v*) and stirred thoroughly to obtain the SA-CMCS-GE complex.

Preparation of cross-linking solution: four concentrations (20 mg/mL, 30 mg/mL, 40 mg/mL, 50 mg/mL) of PMC solution were prepared, four bottles of each concentration. The pH of the same concentration of PMC solution was adjusted to pH2, pH3, pH4 and pH5. CaCl_2_ was added to the 16 solutions to reach 2% concentration.

Simulated gastric and intestinal fluids are shown in Table 1 [42]. Gastric protease (200 U/mL) and gastric mucin (1.5 g/L) were dissolved in simulated gastric fluid (SGF) before digestion. Pancreatin (90 U/mL) and bile salt (5 g/L) were dissolved in simulated intestinal fluid (SIF) before digestion. Finally, SGF and SIF were heated in a 37 °C water bath for 20 min to stimulate enzyme activity.

### 3.4. Preparation of Prunus mume Concentrate Probiotic Microcapsules

The activated *L. plantarum* was centrifuged for 6 min at 4 °C and 6000 rpm to collect the strain. The strain was washed twice with an equal amount of 0.85% (*m*/*v*) physiological saline and resuspended to form a bacterial suspension. The bacterial suspension was added to the SA-CMCS-GE complex (1:9) and was stirred gently for 5 min at 37 °C. The 0.4 mm syringe with a capacity of 1 mL was used to drop the mixed solution (approximately 10 μL/drop) into 16 different cross-linking solutions at a fixed height of about 10 cm from the liquid surface. The microcapsules were cured for 30 min, filtered to obtain the microcapsules, and washed three times with sterile water to obtain *Prunus mume* concentrate probiotic microcapsules.

### 3.5. Macroscopic Observation and Diameter Measurement of Microcapsules

Photos of microcapsules were taken. The diameter of microcapsules was measured by a calibrated digital caliper (resolution 0.01 mm). Fifty microcapsules prepared from each cross-linking solution were randomly selected for diameter measurement, and the average diameter of the microcapsules was calculated.

### 3.6. Swelling Ratio Measurement of Microcapsules in Simulated Digestive Fluid

The swelling ability of microcapsules was evaluated by measuring their swelling changes in simulated digestive fluids. Referring to previous research methods and with slight modifications, the swelling ratio curves of microcapsules were measured in SGF (pH2.5) and SIF (pH7) [43]. The freeze-dried microcapsules (50 mg) were placed in 50 mL of simulated digestion fluid, incubated for 3 h at 37 °C. At the designated time, the microcapsules were removed from the simulated digestive fluid, completely dried with a tissue to remove excess surface water, and then weighed. The swelling ratio of microcapsules was calculated at each time point according to Formula (1).

(1) $$Swelling\;ratio(g/g)=\left(S1-S0\right)/S0$$

*S*1: the weight of microcapsules after swelling at each digestion time point; *S*0: the initial weight of microcapsules. The swelling ratio is dimensionless.

### 3.7. Determination of Encapsulation Efficiency of Probiotic and Organic Acid in Microcapsules

The method of Lemos et al. (2021) was referenced for determining the encapsulation efficiency (EE) of microcapsules [44]. Microcapsules (2.0 g) were dissolved in 18.0 mL sterile sodium citrate solution (10%), and the probiotic and organic acid were released at 240 rpm and 37 °C for 30 min. Then the mixed system was centrifuged at 4 °C and 6000 rpm for 6 min, resuspended in an equal amount of physiological saline. The viable bacteria were counted by using the plate colony counting method. The encapsulation efficiency of microcapsules was calculated according to the formula 2.

(2) Encapsulation efficiency(%)=(E×10/E0)×100

*E*: the number of live bacteria encapsulated in the microcapsules; *E*0: the number of viable bacteria in the bacterial suspension used for preparing microcapsules.

The mixed system was filtered through a 0.22 μm membrane to remove bacteria, resulting in a liquid for detecting organic acid (malic acid, citric acid and lactic acid) content. Determination of organic acid is slightly modified from the method by HPLC system (Waters 1525, Waters, Waters Corporation, Milford, MA, USA) with a C18 (5 μm, 250 × 4.6 mm) column (ZORBAX, Agilent, Agilent Technology Co., Ltd., Santa Rosa, CA, USA) at 30 °C [45,46]. The mobile phase consisted of solvent A (0.02 M KH_2_SO_4_) and solvent B (100% CH_3_OH). The gradient conditions were A: B (99:1, v:v); flow rate: 1.0 mL/min; time: 15 min; injection volume: 10 µL. Peaks were detected by measuring absorbance at 210 nm.

(3) Organic acid encapsulation efficiency(%)=(E×10/E0)×100

*E*: the concentration of organic acid encapsulated in the microcapsules; *E*0: the concentration of organic acid in the cross-linking solution used for preparing microcapsules.

### 3.8. Fourier Transform Infrared Spectroscopy (FTIR) Analysis of Microcapsules

According to the experimental method of Xu et al. (2023), freeze-dried microcapsules (1 mg) and dried KBr powder (100 mg) were mixed and pressed into molds [47]. The infrared spectral structure of microcapsules was determined by a Fourier transform infrared spectrometer (Nicolet iS10, Thermo Fisher Scientific, Waltham, MA, USA), with a wavelength ranging from 400 to 4000 cm^−1^ and a resolution of 4 cm^−1^, with 64 scans.

### 3.9. X-Ray Diffraction (XRD) of Microcapsules

Microcapsules were analyzed by X-ray diffraction (D8 Advance, Bruker, Karlsruhe, Germany). The scanning speed is 4°/min, and the scanning angle (2θ) ranges from 5° to 80°.

### 3.10. Thermogravimetric Analysis (TGA) of Microcapsules

Microcapsules (5 mg) were weighed and placed in a ceramic crucible. Microcapsules were heated from 25 °C to 600 °C at a rate of 10 °C/min under a nitrogen atmosphere (SDTA851E Thermogravimetric Analyzer, METTLER TOLEDO, Greifensee, Switzerland). The thermogravimetric (TG) curve was differentiated to obtain a derivative thermogravimetric (DTG) curve, which was then smoothed using the adjacent averaging method.

### 3.11. Scanning Electron Microscopy (SEM) of Microcapsules

Microcapsules were pre-frozen at −20 °C for 24 h, freeze-dried by a freeze dryer for 48 h. The microcapsules were fixed on a conductive adhesive plate and subjected to gold spraying treatment. The sample was observed under a scanning electron microscope (ZEISS EVO LS10, Carl Zeiss, Oberkochen, Baden-Württemberg, Germany) with an acceleration voltage of 3 kV.

### 3.12. The Resistance of Probiotics in Microcapsules in Simulated Gastric and Intestinal Fluids

The previous method was used to investigate the resistance of probiotics in microcapsules in simulated gastric and intestinal fluids [47]. Microcapsules (1.0 g) prepared according to method 2.4, unencapsulated *L. plantarum* (1.0 mL) was mixed with 9.0 mL of SGF separately, digested at 37 °C and 180 rpm for 3 h. At 0, 1.5, and 3 h of simulated gastric digestion, the SGF was centrifuged at 6000 rpm for 6 min, resuspended in physiological saline, and the number of unencapsulated probiotics was counted. After the microcapsules were removed from the simulated digestion gastric fluid, the microcapsules were dissolved in 9.0 mL sterile sodium citrate solution (10%), and the probiotics were released at 240 rpm and 37 °C for 30 min, followed by live bacterial counting. The method for determining the resistance of probiotics in microcapsules in SIF was the same as that of SGF.

### 3.13. Released Viable Count of Probiotics from Microcapsules in Simulated Digestive Fluid

Referring to the method of Zhu et al., (2024), with slight modifications, the release behavior of microcapsules was evaluated by simulating the human digestive system with gastric fluid for 3 h and intestinal fluid for 3 h [48]. Microcapsules (1.0 g) were mixed with SGF (9.0 mL, pH 2.5), digested at 37 °C and 180 rpm for 3 h. The release of microcapsules was counted at 1, 2, and 3 h. The method for determining the release of microcapsules in SIF was the same as that of SGF.

### 3.14. Statistical Analyses

All data are expressed as the mean values ± standard deviations (SD). Statistical analysis followed by Duncan’s test at *p* < 0.05, using SPSS 25.0 software (Statistical Product and Service Solutions, Armonk, NY, USA). Drawing with Origin 2025 (OriginLab, Northampton, MA, USA).

## 4. Conclusions

This study provides a green and effective strategy for probiotic protection using natural plant concentrates and dual cross-linking, with potential applications in functional food development. The experimental results indicated that the concentration of PMC has little effect on the performance of polysaccharide microcapsules. The microcapsules formed by the dual cross-linking of H^+^ provided by PMC had a smaller diameter. However, low pH may have adverse effects on probiotics, leading to a decrease in EE. FTIR and SEM results confirmed that acid-enhanced hydrogen bonding and electrostatic interactions within polysaccharides, resulting in a more stable structure and optimizing the structural density of microcapsules. During the simulated digestion process, microcapsules achieved precisely controlled release (gastric low release, intestinal targeted release), due to the low swelling of polysaccharides in SGF and high swelling in SIF. The number of viable bacteria in microcapsules significantly increased, increasing by 1 log(CFU/mL) in SGF and 1.25 log(CFU/mL) in SIF, respectively. The composite material of polysaccharides and proteins, as well as regulating natural plant organic acids and Ca^2+^ dual cross-linking construction of microcapsules in this study, lays the foundation for future research on directly using natural plant organic acids to prepare microcapsules, and provides a reference for the study of microcapsules constructed through the dual cross-linking of natural plant concentrates and Ca^2+^ in enhancing the resistance of probiotics during human digestion.

From a clinical and translational standpoint, the dual cross-linking platform based on *Prunus mume* concentrate and Ca^2+^ provides a safe, scalable, and food-grade strategy for oral probiotic delivery. The markedly improved gastric survival and intestinal-targeted release are relevant for clinical conditions where gastrointestinal transit resistance is essential, such as antibiotic-associated diarrhea, irritable bowel syndrome, and inflammatory bowel disease. This technology can be incorporated into functional foods, nutraceuticals, or pharmaceutical formulations. The natural, clean-label composition may enhance patient compliance and facilitate regulatory approval. This approach gives clinicians and scientists a ready template for designing next-generation probiotics, with clinical trials needed to confirm efficacy and safety in patients.

## Figures and Tables

**Table 1:** Average diameter of microcapsules.

Size (mm; n = 50)	pH2	pH3	pH4	pH5	CK
20 mg/mL	1.92 ± 0.12 ^b^	2.01 ± 0.18 ^b^	2.12 ± 0.22 ^a^	2.28 ± 0.26 ^a^	2.15 ± 0.29 ^a^
30 mg/mL	1.87 ± 0.19 ^bc^	1.98 ± 0.19 ^b^	2.18 ± 0.23 ^a^	2.26 ± 0.25 ^a^	2.15 ± 0.29 ^a^
40 mg/mL	1.84 ± 0.13 ^c^	1.99 ± 0.16 ^b^	2.17 ± 0.25 ^a^	2.25 ± 0.27 ^a^	2.15 ± 0.29 ^a^
50 mg/mL	1.83 ± 0.17 ^c^	2.05 ± 0.13 ^ab^	2.12 ± 0.28 ^a^	2.25 ± 0.24 ^a^	2.15 ± 0.2 ^b^

Note: Lowercase letters indicate significant differences (*p* < 0.05) between microcapsules prepared in cross-linking solutions with different pH values at the same PMC concentration and the blank group. CK: control without PMC.

**Table 2:** Preparation of simulated digestive fluid.

	SGF (pH2.5)	SIF (pH7)
	Concentration (mM/L)	Concentration (mM/L)
KCl	6.9	6.8
KH_2_PO_4_	0.9	0.8
NaHCO_3_	25	85
NaCl	47.2	38.4
MgCl_2_·6H_2_O	0.1	0.33
(NH_4_)_2_CO_3_	0.5	-
CaCl_2_	0.15	0.6

Note: indicated the Ca^2+^ concentration in the final simulated digestive fluid. CaCl_2_ solution was not directly added to the simulated digestion solution because it would form a precipitate if left to stand for a long time. Concentrate the simulated digestion solution 1.25 times and prepare it in 400 mL of distilled water. Before use, add a high concentration of CaCl_2_ solution to dilute the simulated digestion solution and achieve the desired concentration of Ca^2+^.

## Data Availability

The original contributions presented in this study are included in the article. Further inquiries can be directed to the corresponding author.
